# Exogenous Lipocalin 2 Ameliorates Acute Rejection in a Mouse Model of Renal Transplantation

**DOI:** 10.1111/ajt.13521

**Published:** 2015-11-23

**Authors:** M. I. Ashraf, H. G. Schwelberger, K. A. Brendel, J. Feurle, J. Andrassy, K. Kotsch, H. Regele, J. Pratschke, H. T. Maier, F. Aigner

**Affiliations:** ^1^ Department of VisceralTransplant and Thoracic SurgeryMedical University InnsbruckInnsbruckAustria; ^2^ Department for General, Visceral and Transplantation Surgery, Campus Virchow‐KlinikumCharité UniversitätsmedizinBerlinGermany; ^3^ Institute of PathologyMedical University InnsbruckInnsbruckAustria; ^4^ Department of Surgery, Clinic GrosshadernLudwig‐Maximilian‐University MunichMunichGermany

**Keywords:** basic (laboratory) research, science, kidney transplantation, nephrology, animal models: murine, rejection: acute

## Abstract

Lipocalin 2 (Lcn2) is rapidly produced by damaged nephron epithelia and is one of the most promising new markers of renal injury, delayed graft function and acute allograft rejection (AR); however, the functional importance of Lcn2 in renal transplantation is largely unknown. To understand the role of Lcn2 in renal AR, kidneys from Balb/c mice were transplanted into C57Bl/6 mice and vice versa and analyzed for morphological and physiological outcomes of AR at posttransplantation days 3, 5, and 7. The allografts showed a steady increase in intensity of interstitial infiltration, tubulitis and periarterial aggregation of lymphocytes associated with a substantial elevation in serum levels of creatinine, urea and Lcn2. Perioperative administration of recombinant Lcn2:siderophore:Fe complex (rLcn2) to recipients resulted in functional and morphological amelioration of the allograft at day 7 almost as efficiently as daily immunosuppression with cyclosporine A (CsA). No significant differences were observed in various donor–recipient combinations (C57Bl/6 wild‐type and Lcn2^−/−^, Balb/c donors and recipients). Histochemical analyses of the allografts showed reduced cell death in recipients treated with rLcn2 or CsA. These results demonstrate that Lcn2 plays an important role in reducing the extent of kidney AR and indicate the therapeutic potential of Lcn2 in transplantation.

## Abstract 2

The authors demonstrate the function of lipocalin 2 as an upregulated marker during acute rejection and as a therapeutic agent to reduce acute rejection in a murine kidney transplantation model, supporting a possible future application of lipocalin 2 as an early biomarker as well as an immunosuppressive agent in kidney transplantation.

**Abbreviations**
AKI: acute kidney injury
AR: allograft rejection
CsA: cyclosporine A
Gr‐1: granulocyte receptor 1
HPF: high‐power field
IRI: ischemia–reperfusion injury
Lcn2: lipocalin 2
PALA: periarterial lymphocytic aggregates
PCR: polymerase chain reaction
rLcn2: recombinant lipocalin 2:siderophore:Fe complex
sCr: serum creatinine
sLnc2: serum lipocalin 2
sUrea: serum urea
TUNEL: terminal transferase‐mediated dUTP nick end labeling

## Introduction

The main problems in kidney transplantation for end‐stage renal disease are acute allograft rejection (AR) and long‐term graft survival, which depend largely on ischemia–reperfusion injury (IRI) of the graft in association with innate and adaptive immune responses mounted by the host 1, 2, 3, 4. Despite advances in both organ preservation and immunosuppressive regimens that have significantly prolonged short‐term graft survival, overall long‐term graft survival has not dramatically changed in recent decades 5. Acute AR, among others, remains the most important risk factor for later chronic rejection and ultimate graft loss, especially when AR occurs in repeated episodes 5, 6, 7, 8. Development of novel strategies that prevent early damage and AR are key to ultimate graft survival 9.

Lipocalin 2 (Lcn2), also known as neutrophil gelatinase‐associated lipocalin, is a 24‐kDa glycoprotein primarily expressed and secreted by neutrophils 10, 11, 12, 13. Lcn2 plays a crucial role in antibacterial innate immune responses by virtue of its ability to bind iron siderophores 14, 15. In addition, its role in growth, differentiation and apoptosis of mammalian cells has been demonstrated and depends largely on regulation of iron homeostasis of the target cells 16, 17, 18, 19.

Moreover, in cases of acute kidney injury (AKI), Lcn2 is strongly expressed in epithelia of damaged nephrons and is released into body fluids. Lcn2 levels in blood serum and urine serve as a promising early marker of AKI and delayed graft function 20, 21, 22. Lcn2 was also proposed to be a sensitive biomarker of acute renal graft rejection during the early posttransplant period and more accurately predicted histologically proven renal AR 23, 24. Although Lcn2 has been evaluated as a biomarker of delayed graft function 20, 21, 22 and AR 23, 24, its mode of action remains to be determined. At present, a discrepancy exists regarding whether Lcn2 attenuates or increases IRI. Recombinant Lcn2 was rapidly taken up by proximal tubuli and recovered renal structure and function in a mouse model of IRI by reducing tubular cell death and enhancing proliferation of the tubular epithelial cells 25, 26. In contrast, the lack of Lcn2 in *Lcn2*‐deficient mice or Lcn2 blockade with anti‐Lcn2 antibodies did not aggravate the ischemia–reperfusion–induced renal injury 27, 28. Using a mouse heterotopic heart transplantation model, we previously proposed a chemoattractant function for Lcn2 in the initiation of inflammatory responses during ischemia–reperfusion 10, 29; however, no experimental data exist about the function of Lcn2 in the course of AR.

Using a mouse model of renal transplantation (Balb/c to C57Bl/6 WT or Lcn2^−/−^ and vice versa), we investigated the role of Lcn2 in renal AR. We present evidence that although endogenously expressed Lcn2 appears to have little effect, perioperative administration of recombinant Lcn2 in the form of recombinant Lcn2:siderophore:Fe complex (rLcn2) significantly reduced allograft damage and improved allograft function.

## Material and Methods

### Animals

Lcn2^−/−^ animals were obtained from Thorsten Berger (Princess Margaret Hospital, Toronto, Canada). WT C57BL/6 animals (H2b) and Balb/c (H2d) mice were purchased from Harlan‐Winkelmann (Harlan Laboratories Srl, Udine, Italy). For our experiments, Lcn2^−/−^ mice were backcrossed into the C57BL/6 background for at least 10 generations. Offspring were genotyped by polymerase chain reaction (PCR) of genomic DNA derived from tail clippings. All mice were housed in a specific pathogen‐free environment with unlimited access to water and standard laboratory chow, and experiments were conducted following approval for institutional animal use under Austrian federal law (BMWF‐66.011/0163‐II/3b/2012). Only male mice weighing 24–28 g were used in this study.

### Mouse renal transplantation

Renal transplantations were performed under inhalation anesthesia with isoflurane (Abbott GmbH, Vienna, Austria), as described previously 30. Following a midline abdominal incision, the left kidney, aorta and inferior vena cava of the donor were fully exposed and mobilized by carefully cauterizing and cutting the small vessels including the left lumbar vein, the underlying vascular branches and gonadal vessels emerging from the renal pedicles. The kidney was flushed *in situ* with histidine‐tryptophane‐ketoglutarate solution and procured *en bloc* including the renal vein; the renal artery, along with a small aortic cuff; and the ureter.

Following left nephrectomy of the recipient, the donor kidney was implanted below the level of native renal vessels. End‐to‐side anastomoses between the donor and recipient vessels were performed using 10‐0 nylon sutures (AROSurgical, Newport Beach, CA). In this knotless technique, the last stitches were not tied to the short ends of the proximal or distal tie. Adjusting the tension on the knotless sutures could perfectly control potential bleeding from the anastomosis.

For urinary tract reconstruction, the ureter was directly anastomosed into the bladder using a pull‐through technique. At the entry site of the bladder, the periureteral fat tissue was fixed to the bladder by two or three interrupted stitches using 10‐0 sutures. At the exit site, the redundant ureter was cut to allow the end of the ureter to retract into the bladder. The times of cold and warm ischemia of the graft were maintained at 40 and 30 min, respectively. The contralateral native kidney was removed 24 h before the allograft harvest to monitor the effect of AR on graft function. Animals with histologically proven technical complications were excluded from the study.

In the rLcn2 treatment group, rLcn2 (250 μg) was applied to the recipients perioperatively 1 h before transplantation, at the time of reperfusion and 1 h after reperfusion. In the immunosuppression group, 10 mg/kg body weight of CsA was subcutaneously administered daily to the recipients.

### Preparation of rLcn2

Mouse Lcn2 without the signal peptide (NP_032517) 31 was expressed and purified as a glutathione S‐transferase fusion protein in *Escherichia coli* BL21, as described previously 29.

### RNA isolation, cDNA synthesis, and quantitative reverse transcription **PCR**

Total RNA was isolated from snap‐frozen mouse kidney tissues using the RNeasy Mini Kit (Qiagen, Hilden, Germany), following the manufacturer's instructions. For cDNA synthesis, 2 µg RNA was reverse transcribed using oligo(dT) primer and RevertAid H Minus M‐MuLV Reverse Transcriptase (Fermentas GmbH, St. Leon‐Rot, Germany). Quantitative reverse transcription PCR was performed with the ABI PRISM 7500 Sequence Detection System using primers designed with Primer Express Software (Life Technologies, Darmstadt, Germany). The data were normalized to the housekeeping gene hypoxanthine‐guanine phosphoribosyltransferase (*HPRT*).

### Immunoblotting

Total protein was precipitated from the flow through of the RNA spin column by adding an equal volume of 100 mM ZnCl_2_ and dissolved in 8 M urea containing 50 mM dithiothreitol. Immunoblotting was performed, as described previously 32, with antibodies specific for cleaved caspase 3 (9664; Cell Signaling Technology, Boston, MA). Antibody complexes were visualized using ECL (Amersham, Buckinghamshire, UK) and quantified by using ImageJ software (National Institutes of Health, Bethesda, MD).

### Assessment of renal function

Renal function was assessed by serum creatinine (sCr), serum urea (sUrea) and serum Lcn2 (sLcn2) measurements. Creatinine and urea were measured using the CREP2 Creatinine Plus version 2 and Urea/BUN assays, respectively, on Roche/Hitachi cobas c 701/702 systems (Roche Diagnostics, Mannheim, Germany), and Lcn2 levels were determined using the Quantikine enzyme‐linked immunosorbent assay kit (R&D Systems, Minneapolis, MN).

### Histopathology, immunohistochemistry, and TUNEL staining

Kidney samples were fixed in buffered formalin and embedded in paraffin following standard procedures. Histology tissue sections (4 μm) were stained with hematoxylin and eosin or periodic acid–Schiff stain, and lesions were scored according to the definitions of Banff classification 33. In addition to the defined lesions, we semiqunatitatively graded periarterial lymphoctic aggregates (PALA; grade 0: absent; grade 1/mild: less prominent PALA visible at intermediate to high magnification; grade 2/moderate: intermediate between grades 1 and 3; grade 3/severe: numerous prominent PALA around arteries visible at scanning view) and acute tubular injury (grade 0: absent; grade 1/mild: tubular ectasia and focal cell detachment; grade 2/moderate: intermediate between grades 1 and 3; grade 3/severe: overt tubular epithelial cell necrosis involving entire tubular cross‐sections).

Immunohistochemical staining was performed, as described previously 10, with the following primary antibodies: Lcn2 (provided by M. Nilsen‐Hamilton, Iowa State University, Ames, IA), cleaved caspase 3 (9664; Cell Signaling Technology), CD3 (A‐0452; Dako, Glostrup, Denmark), CD4 (SAB4503583; Sigma‐Aldrich, St. Louis, MO), CD8 (bs‐0648R; Bioss, Woburn, MA), granulocyte receptor 1 (Gr‐1; MAB1037; R&D Systems). Terminal transferase‐mediated dUTP nick end labeling (TUNEL) was performed using the In Situ Cell Death Detection Kit, POD (Roche, Vienna, Austria), according to the manufacturer's instructions. For each slide, the number of immunostained or TUNEL‐positive cells was determined in three nonoverlapping high‐power fields (×400), and the mean value was calculated for comparisons.

### Statistical analysis

Statistical analyses were performed using the IBM SPSS Statistics 21 software package (IBM, Armonk, NY). Group differences for scale data such as cell counts or expression levels were analyzed with the Kruskal–Wallis test, and differences between individual groups were determined using the Mann–Whitney test. Ordinal data such as pathological Banff scores were analyzed with the chi‐square test. A p‐value <0.05 was considered significant.

## Results

### Development of renal allograft rejection and dysfunction correlates with Lcn2 expression

**Figure 1:**
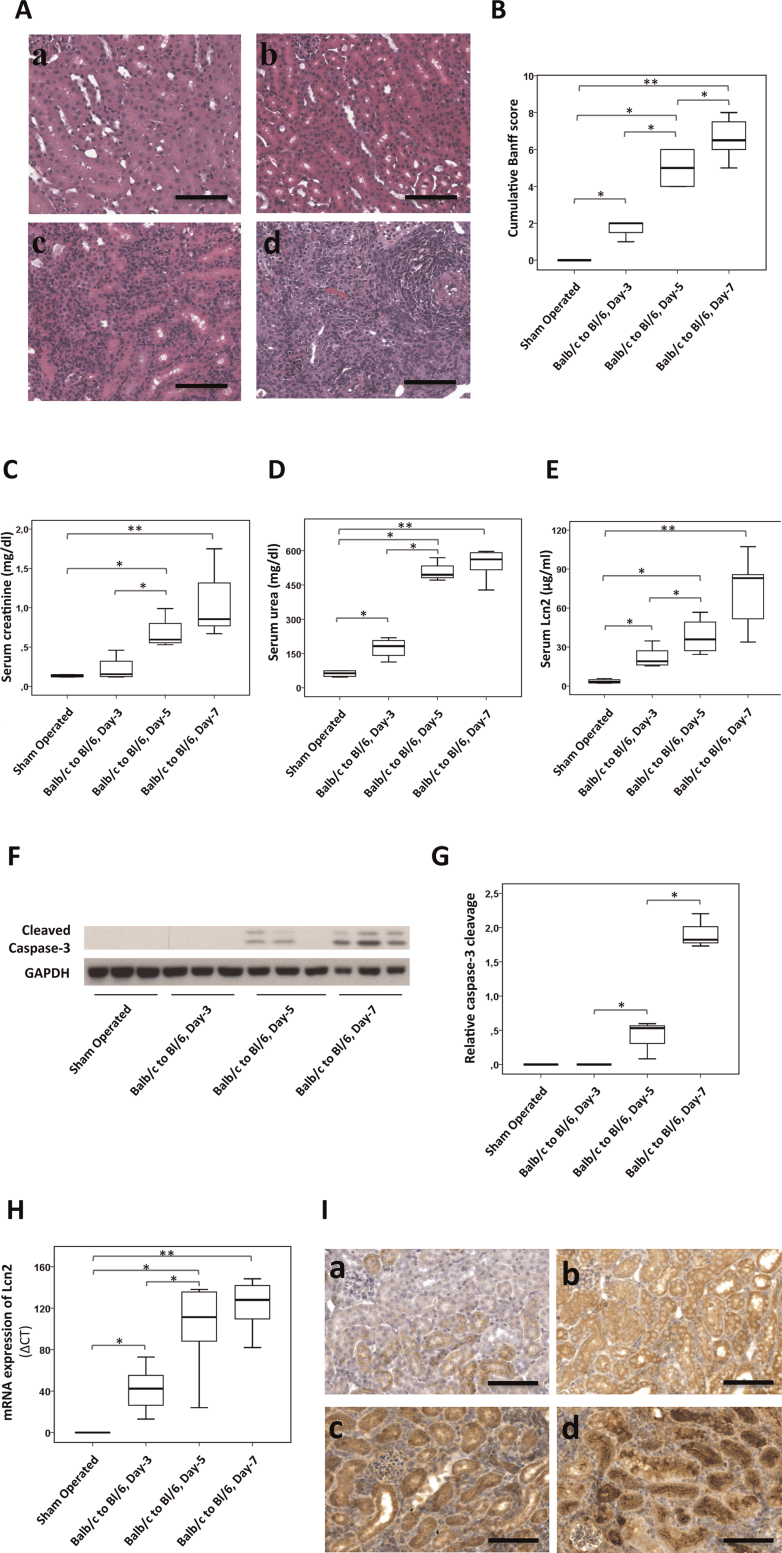
**Kinetics of mouse renal allograft rejection, function, cell death, and lipocalin 2 (Lcn2) expression. ** Kidneys from Balb/c mice were transplanted into C57Bl/6 (Bl/6) mice. Allografts were harvested at posttransplant day 3, 5, or 7, stained with hematoxylin and eosin or periodic acid–Schiff stain and analyzed for histopathology by using Banff criteria. (A) Representative hematoxylin and eosin–stained images of control kidneys from sham‐operated mice (a) and allografts harvested at day 3 (b), day 5 (c), and day 7 (d) are shown. (B) The cumulative Banff score of histological lesions of the allografts harvested at given times is presented by a box plot. Function of the renal allografts was assessed by measurement of serum creatinine (C), serum urea (D), and serum Lcn2 (E) levels. Total kidney lysates of three animals in each of the indicated groups were used to determine activation (cleavage) of caspase 3 by immunoblotting. Representative immunoblot (F) and box plot (G) are shown. Lcn2 mRNA and protein expressions were investigated by quantitative polymerase chain reaction (H) and by immunohistochemistry (I), respectively, in the kidneys of sham‐operated mice (a) and allografts harvested at day 3 (b), day 5 (c), and day 7 (d). n = 6 (allografts harvested at day 3 or 5), n = 8 (allografts harvested at day 7). Scale bars = 100 μm. *p < 0.05, **p < 0.01.

Using a mouse model of renal transplantation (Balb/c to C57Bl/6), we established the kinetics of AR. As shown in Figure 1A and Table 1, cell infiltration was sparse at day 3 but was significantly increased at day 5 and was highest in the allografts harvested at posttransplant day 7, with moderate levels of tubulitis and PALA. Glomerulitis was absent in all of the allografts, and venulitis was rarely observed only in day 7 allografts. The cumulative Banff score of the histological lesions reflected a similar pattern of steady increase in AR over the period of 7 days (Figure 1B). Allograft function was assessed by measuring levels of sCr, sUrea and sLcn2, which also showed a consistent increase over the course of 7 days (Figure 1C–E). Although the first significant increase in sCr was observed at posttransplant day 5, a significant rise in sLcn2 was already detected at day 3.

**Table 1:** Histopathological evaluation of the mouse renal allografts at different posttransplant days using standard Banff classification

Histological lesions	Sham operated (n = 6)	Balb/c to Bl/6 day 3 (n = 6)	Balb/c to Bl/6 day 5 (n = 6)	Balb/c to Bl/6 day 7 (n = 8)
Interstitial inflammation	0	0.6 ± 0.27^a^	2.6 ± 0.24^a,b^	2.75 ± 0.16^a,b^
Tubulitis	0	0	1 ± 0^a,b^	1.88 ± 0.23^a,b,c^
Periarterial lymphocytic aggregates	0	0.4 ± 0.27	1.4 ± 0.24^a^	1.75 ± 0.25^a^

Banff score 0–3: 0, none; 1, <25%; 2, 25–50%; 3, >50%. Glomerulitis and venulitis scores were 0 in most cases; therefore, they were not included.

^a^ Significantly different from group sham operated, ^b^Balb/c to Bl/6 day 3, ^c^Balb/c to Bl/6 day 5, Pearson chi‐square test, p < 0.05.

Because lymphocyte‐mediated apoptotic cell death of the allograft is a hallmark of AR 34, 35, we analyzed cell death in the allograft by immunoblotting tissue lysates with antibodies specific for activated caspase 3 (Figure 1F and G). Caspase 3 activation was observed at posttransplant day 5, with a further increase at day 7. Similar effects were observed when cell death was analyzed by immunohistochemical analysis of activated caspase 3 and TUNEL assay (data not shown).

Lcn2 expression has been shown to correlate with AR in human biopsies 23. We also observed a significant upregulation of Lcn2 mRNA at day 3, with further increases at days 5 and 7 (Figure 1H). At the protein level, Lcn2 expression also increased accordingly, with the strongest upregulation seen in the allografts harvested at posttransplant day 7. Lcn2 staining was observed predominantly in the proximal tubules and in a few infiltrating cells (Figure 1I). Table 2 demonstrates the correlation of Lcn2 mRNA expression in the allograft and sLcn2 with renal functional markers (sCr and sUrea) and Banff score of histology. The increase in Lcn2 mRNA and sLcn2 levels over the course of 7 days correlated strongly with the increase in sCr, sUrea and Banff score of the allografts.

**Table 2:** Correlation of lipocalin 2 (Lcn2) mRNA in the allograft and serum Lcn2 concentration with renal function parameters and the histological lesions following allogeneic kidney transplantation in mouse

	Lcn2 mRNA		Serum Lcn2	
Kidney function indicators	r^2^	p‐value	r^2^	p‐value
Serum creatinine	0.845	0.040	0.996	0.001
Serum urea	0.988	0.003	0.86	0.036
**Histology lesions**				
Interstitial infiltrate	0.976	0.006	0.87	0.035
Tubulitis	0.780	0.058	0.998	0.0003
Periarterial lymphocytic aggregates	0.984	0.004	0.882	0.030
Cumulative Banff score	0.955	0.011	0.930	0.018

### Role of Lcn2 in renal AR

**Figure 2:**
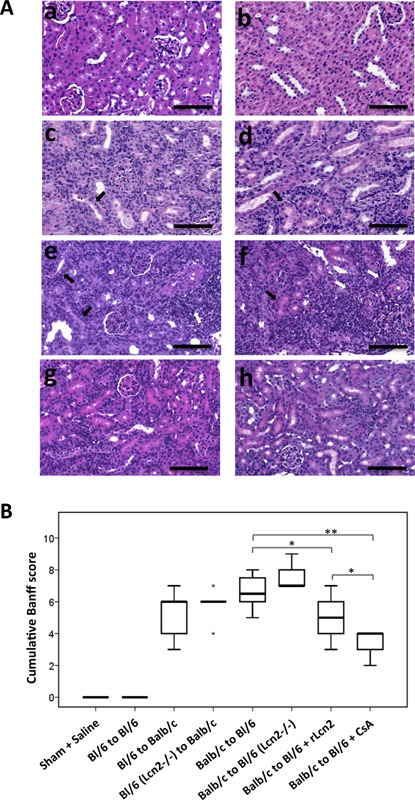
**Effect of lipocalin 2 (Lcn2) on histopathology of the mouse allografts. ** (A) Representative images of hematoxylin and eosin–stained sections of the kidneys from sham‐operated mice (a), C57Bl/6 (Bl/6) kidney isografts (b) and the kidney allografts Bl/6 to Balb/c (c), Bl/6 Lcn2^−/−^ to Balb/c (d), Balb/c to Bl/6 (e), Balb/c to Bl/6 Lcn2^−/−^ (f), Balb/c to Bl/6 (treated with recombinant Lcn2:Sid:Fe complex (rLcn2; 250 μg, perioperatively) (g), Balb/c to Bl/6 (treated with cyclosporine A [10 mg/kg body weight, daily]) (h), harvested at posttransplant day 7 are shown. Black and white arrows highlight the examples of tubulitis and periarterial lymphocytic aggregates, respectively. (B) The cumulative Banff score of the prominent histopathological lesions of the kidney allografts is presented by a box plot (n = 6, except the groups Balb/c to Bl/6 and Balb/c to Bl/6 + rLcn2 n = 8). Scale bars = 100 μm. *p < 0.05, **p < 0.01.

To investigate whether Lcn2 ameliorates or aggravates AR, we transplanted C57Bl/6 WT and Lcn2^−/−^ kidneys into Balb/c recipients and vice versa and analyzed histological changes at posttransplant day 7. The allografts showed moderate to severe tubulointerstitial rejection, as evident by strong infiltration of lymphocytes in the interstitial and periarterial regions, with obvious signs of tubulitis (Figure 2A and Table 3). Likewise, cumulative Banff score of the histological lesions was also high in these allografts (Figure 2B). C57Bl/6 allografts showed reduced PALA compared with the Balb/c allografts (Figure 2A[c,e] and Table 3). The cumulative Banff score and the individual histological lesions were slightly higher in the Lcn2^−/−^ recipients compared with the WT recipients, although not significantly different (Table 3 and Figure 2B).

**Table 3:** Histopathological evaluation of the renal allografts from different treatment groups at posttransplant day 7 by using standard Banff classification

Histological lesions	Sham operated, saline (n = 6)	Bl/6 to Bl/6 (n = 6)	Bl/6 to Balb/c (n = 6)	Bl/6 (Lcn2^−/−^) to Balb/c (n = 6)	Balb/c to Bl/6 (n = 8)	Balb/c to Bl/6 (Lcn2^−/−^) (n = 6)	Balb/c to Bl/6, rLcn2 (250 mg) (n = 8)	Balb/c to Bl/6, CsA (10 mg/kg) (n = 6)
Interstitial inflammation	0	0	2.67 ± 0.23	2.83 ± 0.18	2.75 ± 0.16	2.8 ± 0.2	2.2 ± 0.22^a^	1.8 ± 0.2^a^
Tubulitis	0	0	1.83 ± 0.34	2.5 ± 0.25	1.88 ± 0.23	2.4 ± 0.25	1.3 ± 0.17^a^	1.2 ± 0.2^a^
Periarterial lymphocytic aggregates	0	0	0.83 ± 0.18	0.5 ± 0.24	1.75 ± 0.25^b^	2.2 ± 0.37	1.5 ± 0.24	0.4 ± 0.24^a^
Acute tubular injury	0	0.67 ± 0.21	0.80 ± 0.34	0.67 ± 0.21	0.75 ± 0.27	0.70 ± 0.32	0.78 ± 0.15	0.6 ± 0.25

Banff score 0–3: 0, none; 1, <25%; 2, 25–50%; 3, >50%. Glomerulitis and venulitis scores were 0 in most cases; therefore, they were not included. CsA, cyclosporine A; rLcn2, recombinant lipocalin 2:siderophore:Fe complex.

^a^ Significantly different from group Balb/c to Bl/6, ^b^significantly different from group Bl/6 to Balb/c, Pearson chi‐square test, p < 0.05.

Exogenously administered rLcn2 has been shown to ameliorate injury in a mouse model of renal IRI 25. To understand whether supplementation of rLcn2 may also provide a similar benefit to the renal allograft, we treated the C57Bl/6 WT recipients with rLcn2 (250 μg). Interestingly, the interstitial infiltrates and tubulitis were significantly reduced by the treatment (Figure 2A and Table 3). The cumulative Banff score of the histological lesions was also significantly reduced in the group receiving rLcn2 (Figure 2B). Allografts of recipients treated daily with the potent immunosuppressant CsA (10 mg/kg body weight) showed very few infiltrates and PALA, with almost no signs of tubulitis. The acute tubular injury associated with alloantigen‐independent damage of the grafts was mild and similar in the iso‐ and allografts (Table 3 and Figure S1).

### Treatment with rLcn2 rescued mouse renal allograft function

**Figure 3:**
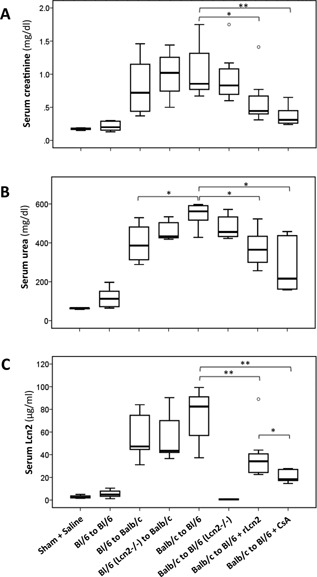
**Amelioration of renal allograft function by peritransplant application of recombinant lipocalin 2 (rLcn2 + Siderophore + Fe). ** Kidneys were transplanted syngenically from C57Bl/6 (Bl/6) to Bl/6 mice and allogenically from Bl/6 WT or Lcn2^−/−^ to Balb/c and Balb/c to Bl/6 WT or Lcn2^−/−^ mice. Iso‐ and allograft function was assessed by measurement of creatinine (A), urea (B), and Lcn2 (C) levels in the serum of the recipients at posttransplant day 7 (n = 6, except the groups Balb/c to Bl/6 and Balb/c to Bl/6 + rLcn2 n = 8). *p < 0.05, **p < 0.01.

Allogeneic kidney transplantation from C57Bl/6 (WT or Lcn2^−/−^) to Balb/c mice and vice versa resulted in substantial elevation in levels of the kidney function markers sCr and sUrea at posttransplant day 7 (Figure 3A and B); however, no significant differences in the sCr and sUrea levels between the Balb/c recipients of C57Bl/6 Lcn2^−/−^ and WT allografts were demonstrated. Likewise, no significant differences in sCr and sUrea were observed between C57Bl/6 Lcn2^−/−^ and WT recipients. Although sLcn2 was hardly detected in sham‐operated animals and C57Bl/6 Lcn2^−/−^ recipients, high sLcn2 levels were observed in C57Bl/6 WT recipients and the Balb/c recipients of C57Bl/6 WT and Lcn2^−/−^ allografts (Figure 3C). Notably, sCr, sUrea and sLcn2 levels in C57Bl/6 recipients treated with rLcn2 were significantly lower than in untreated animals and even lower in mice treated daily with CsA (Figure 3A–C).

### Effects of rLcn2 on renal allograft damage following transplantation

**Figure 4:**
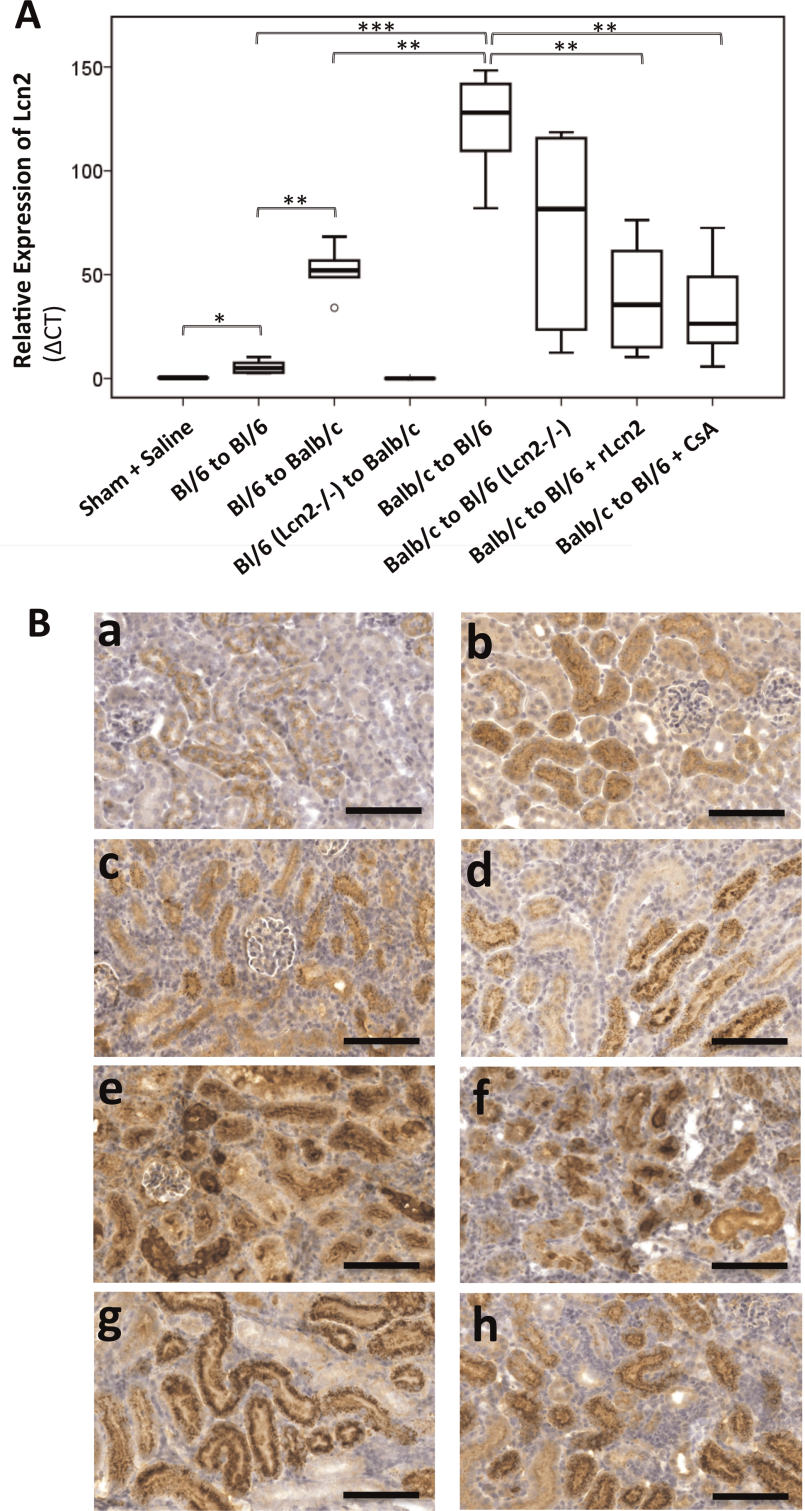
**Effect of recombinant lipocalin 2:siderophore:Fe complex (rLcn2) on the expression of endogenous lipocalin 2 (Lcn2) in the renal allografts. ** Kidneys transplanted from C57Bl/6 (Bl/6) to Bl/6, from Bl/6 to Balb/c and from Balb/c to Bl/6 mice were harvested at posttransplant day 7, and the expression pattern of Lcn2 mRNA and protein in the grafts was determined by quantitative polymerase chain reaction (A) and by immunohistochemistry (B). Representative box plot for mRNA expression (A) and images of the Lcn2 stained (B) sections of kidneys harvested from sham‐operated mice (a), Bl/6 kidney isografts (b), Bl/6 allografts in Balb/c recipients (c), Bl/6 Lcn2^−/−^ allografts in Balb/c recipients (d), and Balb/c allografts in Bl/6 WT (e) and Lcn2^−/−^ (f) recipients and the Bl/6 WT recipients treated with rLcn2 (250 μg perioperatively) (g) or cyclosporine A (10 mg/kg body weight, daily) (h) are shown (n = 6). Scale bars = 100 μm. *p < 0.05, **p < 0.01.

Because Lcn2 expression is a critical indicator of the degree of tissue damage, we tested whether the morphological and physiological benefits achieved by the treatment with rLcn2 were reflected by Lcn2 expression. Although Lcn2 mRNA and protein were hardly detectable in kidneys of sham‐operated animals and the C57Bl/6 Lcn2^−/−^ allografts and were still induced in a syngeneic setting at posttransplant day 7, strong upregulation was observed in the WT allografts (Figure 4A and B). Some granular staining was observed in the tubuli of Lcn2^−/−^ allografts and may be attributed to the uptake of the circulating Lcn2 protein by the tubular cells (Figure 4B[d]). Lcn2 mRNA and protein expression was higher in the Balb/c than C57Bl/6 allografts. The allografts transplanted into the C57Bl/6 Lcn2^−/−^ recipients showed less Lcn2 mRNA and protein expression in comparison to WT recipients. Interestingly, treatment of the C57Bl/6 WT recipients with rLcn2 or CsA significantly reduced Lcn2 mRNA and protein expression in the allografts, consistent with the effects on AR (Figure 2) and function (Figure 3). Lcn2 staining was predominantly localized in tubular cells in the corticomedullary region of the allografts (Figure 4B). The staining pattern in the C57Bl/6 WT recipients treated with either rLcn2 or CsA was granular cytosolic, similar to control kidneys and as expected for a secretory protein stored inside the cells. In contrast, staining in untreated C57Bl/6 WT and Lcn2^−/−^ recipients was mostly diffuse cytosolic and partially extracellular, which might be due to the pronounced destruction of cellular and subcellular structures observed in these groups.

### Loss of renal allograft function may result from cell death in the graft

**Figure 5:**
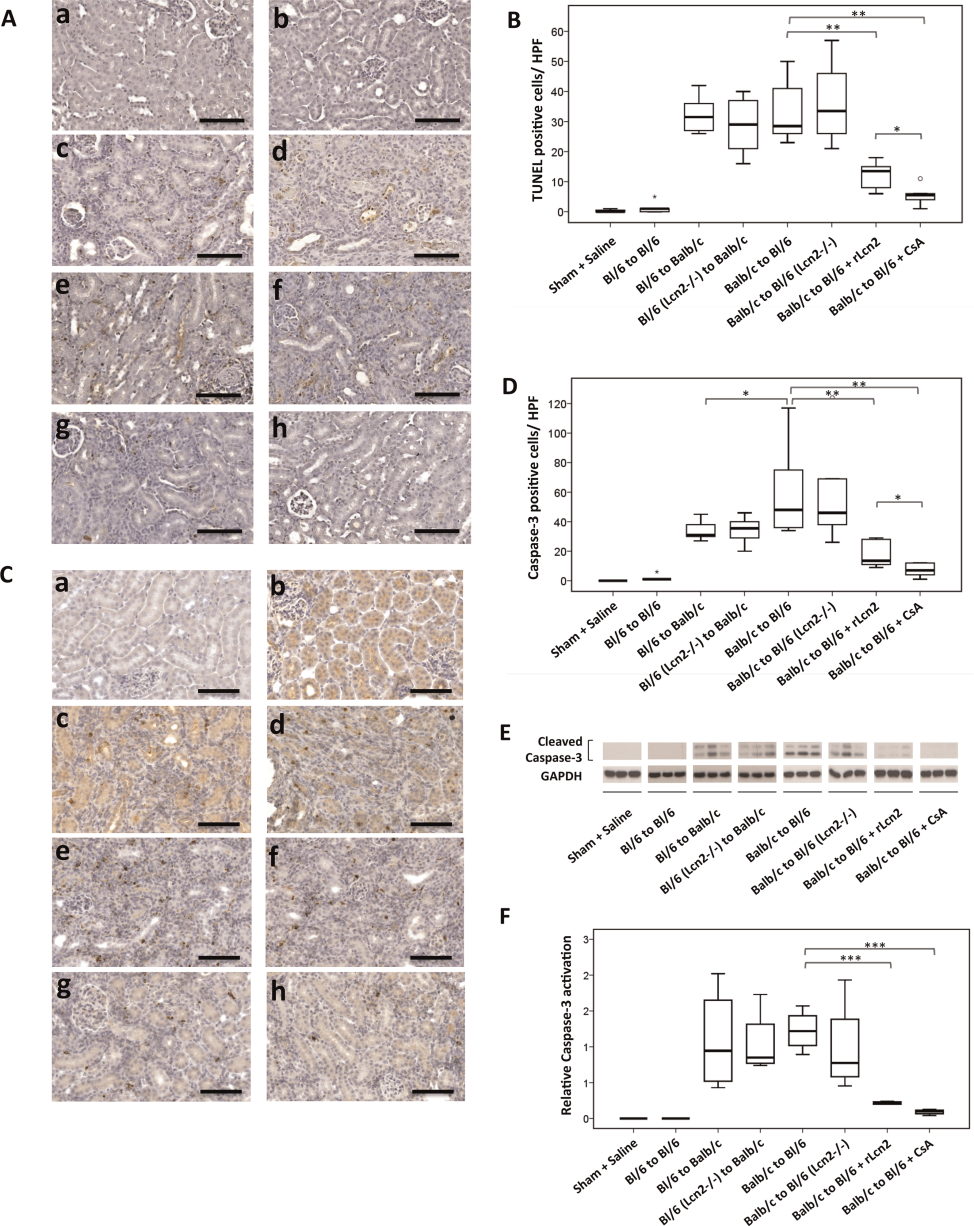
**Recombinant lipocalin 2:siderophore:Fe complex (rLcn2) protects mouse kidney allografts from transplantation‐induced apoptosis. ** Cell death in the C57Bl/6 (Bl/6) isografts, Balb/c kidney allografts transplanted into Bl/6 recipients, and Bl/6 allografts transplanted into Balb/c recipients was determined at posttransplant day 7 by terminal transferase‐mediated dUTP nick end labeling (TUNEL) staining (A, B) for DNA fragmentation and by immunohistochemical analysis (C, D) and immunoblotting (E, F) for caspase 3 activation. Representative images of the TUNEL (A) and activated caspase 3 (C) stained sections of kidneys harvested from sham‐operated mice (a), Bl/6 kidney isografts (b), Bl/6 allografts in Balb/c recipients (c), Bl/6 Lcn2^−/−^ allografts in Balb/c recipients (d), and Balb/c allografts in Bl/6 WT (e) and Lcn2^−/−^ (f) recipients and the Bl/6 WT recipients treated with rLcn2 (250 μg perioperatively) (g) or cyclosporine A (10 mg/kg body weight, daily) (h) are shown (A, C). Quantification of the TUNEL (B) and activated caspase 3 (D)–positive cells per high‐power field are presented by box plots. (E, F) Total kidney lysates of the indicated groups were used to determine activation (cleavage) of caspase 3 by immunoblotting. Representative immunoblots of three animals in each group are shown (E). Relative activation of caspase 3 was determined using glyceraldehyde 3‐phosphate dehydrogenase as a loading control and is presented by a box plot (F). Scale bars = 100 μm. n = 6. *p < 0.05, **p < 0.01, ***p < 0.001.

Kidney transplantation from Balb/c to C57Bl/6 mice and vice versa resulted in considerable cell death in the allografts, evident by a large number of TUNEL‐ and activated caspase 3–positive cells, predominantly in the interstitial regions (Figure 5A–D). There was no significant difference in the numbers of both TUNEL‐ and caspase 3–positive cells between C57Bl/6 WT and Lcn2^−/−^ allografts in Balb/c recipients or between the Balb/c allografts in C57Bl/6 WT and Lcn2^−/−^ recipients. Strikingly, the perioperative administration of rLcn2 into C57Bl/6 WT recipients significantly reduced the frequency of both TUNEL‐ and caspase 3–positive cells in the allografts. The numbers of TUNEL‐ and caspase 3–positive cells were further reduced in allografts of recipients treated daily with CsA (Figure 5B and D). Total tissue lysates of the kidney allografts were also analyzed for processing of caspase 3 by immunoblotting (Figure 5E and F). Caspase 3 cleavage was significantly reduced by treatment of C57Bl/6 WT recipients with rLcn2 or CsA compared with untreated recipients.

### Immunophenotyping of cellular infiltrate of renal allografts

**Figure 6:**
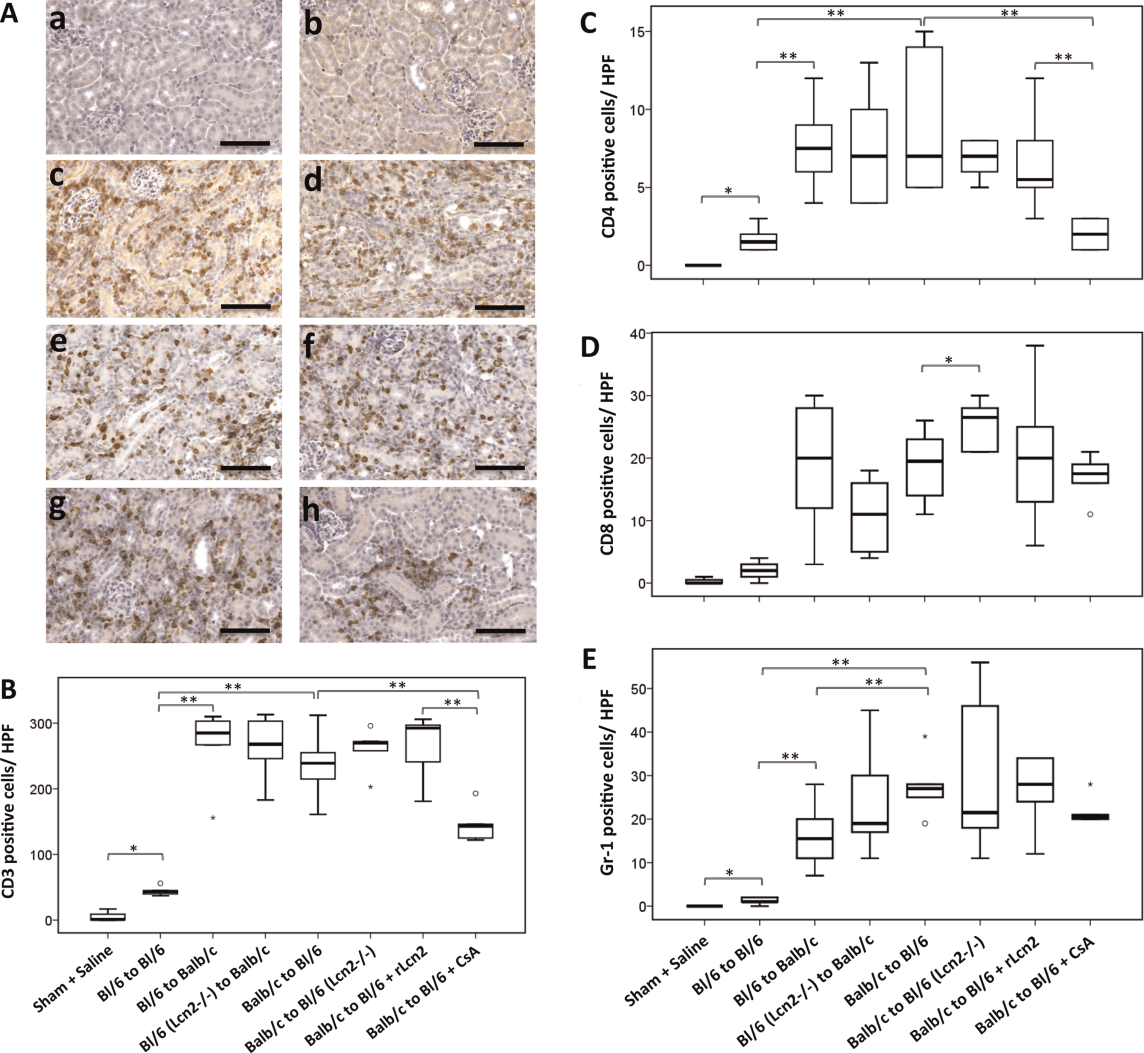
**Immunophenotyping of cells infiltrating mouse renal allografts. ** (A) Representative images of the CD3 stained sections of kidneys harvested from sham‐operated mice (a), C57Bl/6 (Bl/6) kidney isografts (b), Bl/6 allografts in Balb/c recipients (c), Bl/6 Lcn2^−/−^ allografts in Balb/c recipients (d), and Balb/c allografts in Bl/6 WT (e) and Lcn2^−/−^ (f) recipients and the Bl/6 WT recipients treated with recombinant lipocalin 2:siderophore:Fe complex (250 μg perioperatively) (g) or cyclosporine A (10 mg/kg body weight, daily) (h) are shown. Representative box plots of the quantification of CD3 (B), CD4 (C), CD8 (D), and Gr‐1 (E)–positive cells per high‐power field (HPF) are shown. n = 6. Scale bars = 100 μm. *p < 0.05, **p < 0.01.

The allograft infiltrating host cell populations were characterized by immunostaining of tissue sections with antibodies specific for T lymphocyte receptors CD3, CD4 and CD8 and granulocyte receptor Gr‐1. In comparison with the kidneys from sham‐operated mice, allograft tissue sections displayed a large number of CD3^+^ cells (Figure 6A). Quantitative analyses showed no significant difference in the number of CD3^+^ cells in the allografts of Balb/c recipients, in untreated C57Bl/6 (WT and Lcn2^−/−^) recipients and in C57Bl/6 WT recipients treated with rLcn2; however, the number of CD3^+^ cells was significantly lower in the allografts of C57Bl/6 recipients treated with CsA (Figure 6B). Although CD4^+^ cells were infrequent in all allografts, their number was significantly lower in allografts of the recipients treated with CsA (Figure 6C). Although the number of CD8^+^ cells was higher in allografts of C57Bl/6 Lcn2^−/−^ recipients compared with WT, treatment with rLcn2 or CsA had no effect on CD8^+^ cell infiltration (Figure 6D). The number of the infiltrating Gr‐1^+^ cells was generally higher in the Balb/c allografts compared with the C57Bl/6 allografts (Figure 6E). The infiltration of CD3‐, CD4‐ and Gr‐1–positive cells was increased in the C57Bl/6 isografts, although far less pronounced than in the allografts (Figure 6A–C and E).

## Discussion

Although clinical association of Lcn2 expression and AKI has been described extensively in several pathological conditions 20, 21, 22, 23, 24, 36, 37, 38, 39, 40, 41, its role in the course of kidney transplantation is not clear. We investigated the function of Lcn2 in acute AR using a mouse allogeneic kidney transplantation model. Comparing the fate of Balb/c kidney allografts in C57Bl/6 WT and Lcn2^−/−^ recipients or C57Bl/6 WT and Lcn2^−/−^ kidneys in Balb/c recipients, we found no significant differences in histological damage, graft function and apoptosis at posttransplant day 7. One notable difference observed was a slightly elevated sLcn2 concentration in C57Bl/6 Lcn2^−/−^ recipients compared with dramatically increased levels in the WT recipients (Figure 3C). This and the high levels of sLcn2 in the Balb/c recipients of C57Bl/6 Lcn2^−/−^ allografts indicate that in the course of renal rejection, the majority of sLcn2 does not originate from the renal graft, which constitutes the only source of Lcn2 in C57Bl/6 Lcn2^−/−^ recipients, but is predominantly contributed by the host. In contrast, the Lcn2 produced in the damaged renal tubules has been reported to predominantly contribute to urinary Lcn2 42. Likewise, the reduced Lcn2 mRNA and protein detection in the allografts of Lcn2^−/−^ recipients (Figure 4) may be attributed to the lack of Lcn2 in the infiltrating cells because infiltrating polymorphonuclear cells along with tubular epithelial cells have been described as primary sources of Lcn2 in the settings of renal inflammation and transplantation 41, 42, 43, 44. Lcn2 immunoreactivity thus reflects Lcn2 taken up into proximal tubular cells via the endocytic pathway (Figure 4B[d]).

A tendency toward slightly augmented histological lesions of the allograft in C57Bl/6 Lcn2^−/−^ recipients (Figure 2 and Table 3) and concomitantly increased CD8^+^ cells in these grafts (Figure 6D) indicate the existence of some renoprotective and immunoregulatory effect of the Lcn2 expressed by the recipients. Lcn2 has been implicated in differential regulation of immune complex–mediated autoimmune disorders 45. Although Lcn2^−/−^ mice exhibited substantially reduced skin inflammation in the setting of antibody‐mediated acute inflammation, Lcn2^−/−^ mice developed serum‐induced severe arthritis 45.

Treatment of the C57Bl/6 WT recipients with rLcn2 ameliorated histological damage and functional impairment of the allograft almost as efficiently as daily immunosuppression with CsA. The mechanism of renoprotection in this setting is not clear. As demonstrated for renal IRI, Lcn2 ameliorates morphological and functional damage by reducing apoptosis of tubular epithelial cells and stimulating their proliferation, and this apparently depends on the delivery of sufficient amounts of iron to specific cellular structures 25, 26. In a cell model, Lcn2 has been shown to deliver iron and block apoptosis in its iron‐loaded form, whereas it depletes cellular iron and induces apoptosis in its iron‐lacking form 16. A family of common metabolites called *catechols* has also been implicated in high‐affinity binding, effective sequestration and transport of iron to cells of the kidney by Lcn2 46. In addition, Lcn2 might have an acute compensatory, protective role in response to cellular stress through modulating cellular immunity by inducing T cell apoptosis and by upregulation of regulatory T cells 47, 48. Previous publications have suggested a mechanistic link between exogenous Lcn2 and inhibition of caspase 3 activation and thus reduction of renal tubular cell apoptosis and protection of renal function in IRI 49.

Despite the fact that exogenously administered rLcn2 efficiently counteracted allograft damage, endogenous Lcn2 expression and secretion appeared to occur in proportion to the extent of tissue injury and may serve as a marker for allograft damage 50. Endogenous Lcn2 production is probably induced too late to effectively prevent the damage 25, 26. In addition, it is not clear whether endogenously produced Lcn2 appears in its iron‐loaded form and thus would be able to deliver iron to damaged cells. Similar to our findings, a renoprotecive effect of rLcn2 administration in the setting of IRI has been linked to reduced Lcn2 mRNA expression 25.

Mitochondrial permeability transition is a process that induces necrotic cell death dependent on the mitochondrial matrix protein cyclophilin D, an intracellular target of CsA, and the role of this process in our model of AR is still unclear. One might also infer that the mitochondrial permeability transition–blocking potential of CsA would result in reduced inflammatory response, reduced regulated necrosis and better overall graft survival 51, 52. Comparing kidney isografts with allografts indicates, as expected, a mild contribution of alloantigen‐independent IRI because cold and warm ischemia were maintained for a minimum of 40 and 30 min, respectively. Nevertheless, the increased effect of renal IRI on AR is well established and cannot be excluded from these findings 34, 53, 54, although it is hardly detectable at posttransplant day 7 in the isogeneic setting (Figures 2–5).

In this study, we limited our analyses to a follow‐up period of 7 days, in which Lcn2 treatment clearly showed benefit in terms of histological tissue damage and graft function, although it is not clear why particular morphological features (e.g. PALA) differed between allografts of different genetic backgrounds (Table 3). Future studies will have to test the outcome in comparison and in combination with immunosuppression in the long term. Because administration of Lcn2 is effective only at the time of transplantation and the protein is rapidly taken up by the kidneys and secreted in the urine 26, Lcn2 treatment might briefly dampen host immune functions 47, 48 but likely will not compromise the recipient's immune system permanently. The treatment holds great promise because it might eventually replace or at least reduce the requirement for chronic immunosuppression and thus avoid its unfavorable side effects.

## Outlook

Future investigations will be necessary to address the mechanistic effects of Lcn2 on important signaling for cell survival and tissue inflammation (e.g. protein kinase B/mammalian target of rapamycin, extracellular signal‐regulated kinases, nuclear factor‐κB) and on prevention and treatment of AR. It remains unclear why rLcn2 treatment reduces Banff lesions and improves renal function without affecting T cell and neutrophil counts in the graft.

## Disclosure

The authors of this manuscript have no conflicts of interest to disclose as described by the *American Journal of Transplantation*.

## Supporting information

Additional Supporting Information may be found in the online version of this article.
**Supplemental Material and Methods**.

**Figure S1: Histology of the renal grafts**. Kidneys were transplanted syngenically from C57Bl/6 (Bl/6) to Bl/6 mice and allogenically from Bl/6 to Balb/c and Balb/c to Bl/6 mice. The grafts were harvested at posttransplant day 7, stained with hematoxylin and eosin or periodic acid–Schiff and analyzed for acute tubular injury (A) and cast formation (B). The representative box plots are shown.
