# Supplementary material for: Exogenous Lipocalin 2 Ameliorates Acute Rejection in a Mouse Model of Renal Transplantation
Source: Am J Transplant. 2015 Nov 23;16(3):808–20. doi: 10.1111/ajt.13521 (PMC4996417; doi:10.1111/ajt.13521)
Supplement: Supplementary file 1 — Figure S1: Histology of the renal grafts. Kidneys were transplanted syngenically from C57Bl/6 (Bl/6) to Bl/6 mice and allogenically from Bl/6 to Balb/c and Balb/c to Bl/6 mice. The grafts were harvested at posttransplant day 7, stained with hematoxylin and eosin or periodic acid–Schiff and analyzed for acute tubular injury (A) and cast formation (B). The representative box plots are shown. [file AJT-16-808-s001.docx]

**Supplemental Material and Methods**

**Animals**

Lcn2^-/-^ animals were obtained from Thorsten Berger, The Campbell Family Institute for Cancer Research, University of Toronto, Princess Margaret Hospital, Toronto, Canada. Wild-type C57BL/6 animals (Lcn2 wt) (H2b) and Balb/c (H2d) mice were purchased from Harlan-Winkelmann (Harlan Laboratories Srl, Italy). For our experiments Lcn2^-/-^ mice were backcrossed into the C57BL/6 background for at least ten generations. Offsprings were genotyped by PCR of genomic DNA derived from tail clippings. All mice were housed in a specific pathogen-free environment with unlimited access to water and standard laboratory chow, and experiments were conducted following approval by the Institutional Animal Use under Austrian Federal Law (BMWF-66.011/0163-II/3b/2012) and were performed in accordance with national animal protection guidelines. Only male mice, weighing 24-28g, were used in this study.

**Mouse renal transplantation**

Renal transplantations from Balb/c to C57Bl/6 wt or Lcn2^-/-^ mice and vice versa were performed under inhalation anesthesia with isoflurane (Abbott GmbH, Vienna, Austria), as previously described ([1](#_ENREF_1)). Following a midline abdominal incision, the left kidney, aorta and inferior vena cava of the donor were fully exposed and mobilized by carefully cauterizing and cutting the small vessels including the left lumbar vein, the underlying vascular branches and gonadal vessels emerging from the renal pedicles. The kidney was flushed *in situ* with histidine-tryptophane-ketoglutarate (HTK) solution and procured *en bloc* including renal vein, renal artery along with a small aortic cuff and ureter.

Following left nephrectomy of the recipient, the donor kidney was implanted below the level of native renal vessels. End-to-side anastomoses between the donor and recipient vessels were performed using 10-0 nylon sutures (ARO Surgical, CA). In this knotless technique the last stitches were not tied to the short ends of the proximal or distal tie. Adjusting the tension on the knotless sutures could perfectly control potential bleeding from the anastomosis.

For urinary tract reconstruction, the ureter was directly anastomosed into the bladder using a pull-through technique. At the entering site of the bladder the periureteral fat tissue was fixed to the bladder by 2-3 interrupted stiches using 10-0 sutures. At the exit site the redundant ureter was cut to allow the end of the ureter to retract into the bladder. The hole on the right wall of the bladder was closed via a figure of eight stitch using 10-0 suture. The times of cold and warm ischemia of the graft were maintained at 40 and 30 min, respectively. The contralateral native kidney was removed 24 hours before the allograft harvest in order to monitor the effect of AR on its function. Animals with histologically proven technical complications were excluded from the study.

The sham operated mice underwent a surgical procedure similar to the recipients with the exception of renal transplantation. They were given either saline or cyclosporin A (CsA, 10 mg/kg BW) daily for 7 days and, to completely mimic the transplantation groups, left nephrectomy was performed on day 6. In the Lcn2 treatment group, recombinant Lcn2:Siderophore:Fe (rLcn2:Sid:Fe) complex (250 µg) was applied to the recipients perioperatively one hour before transplantation, at the time of reperfusion and one hour post-reperfusion. In the immunosuppression group, 10 mg/kg BW CsA was daily administered subcutaneously to the recipients.

**Preparation of Lcn2:Siderophore:Fe complex**

Mouse Lcn2 cDNA encoding the mature protein without the 20 amino acid signal peptide (NM_008491.1)([2](#_ENREF_2)) was amplified by PCR with specific primers from total mouse uterus cDNA and cloned in frame into the bacterial expression vector pGEX-4T-3 (GE Healthcare, Vienna, Austria). The recombinant plasmid was transformed into the protease deficient strain *E. coli* BL21 to produce a glutathione S-transferase fusion protein according to manufacturer's instructions (GE Healthcare, Vienna, Austria). The fusion protein was purified by chromatography on Glutathione Sepharose (GSTrap FF, GE Healthcare, Vienna, Austria), cleaved with thrombin protease, and Lcn2 was purified to greater than 99% purity by chromatography on CIM-SO_3_ (BIA Separations, Ljubljana, Slovenia). The recombinant Lcn2 protein was incubated for 4 hours at 4°C with an equimolar amount of ferric enterobactin (EMC microcollections, Tübingen, Germany) and dissolved in phosphate buffered saline at 1 mg/ml for in vivo application.

**RNA isolation and cDNA synthesis**

Total RNA was isolated from snap frozen mouse kidney tissues using RNeasy mini kit (Qiagen, Hilden, Germany) following manufacturer’s instructions, which included a DNAse-I digest with RNase-Free DNase set (Qiagen, Hilden, Germany) to avoid contamination of genomic DNA. RNA integrity and quality was verified by agarose gel-electrophoresis and spectrophotometrically, respectively. For cDNA synthesis, 2 μg of total RNA was reverse transcribed in a 40 μl reaction volume using oligo(dT) primer and the RevertAidTM H Minus M-MuLV Reverse Transcriptase (Fermentas GmbH, St. Leon-Rot, Germany).

**Quantitative Real Time (qRT)-PCR**

Real-time reverse transcription polymerase chain reaction (RT-PCR) for gene expression analysis was performed with the ABI PRISM 7500 Sequence Detection System (Life Technologies, Darmstadt, Germany). Primers were designed using Primer Express Software (Life Technologies) and validated. The PCR reaction was performed in a final volume of 25 µl containing 1 µl cDNA, 12,5µl Master Mix (Life Technologies), 1 µl fluorogenic hybridization probe, 6µl primer mix, and 5,5µl distilled water. The amplification consisted of a two-step PCR (40 cycles; 15s denaturation step at 95°C and 1 min annealing/extension step at 60°C). The mean Ct values were calculated from double determinations and samples were considered negative if the Ct values exceeded 40 cycles. Specific gene expression was normalized to the housekeeping gene hypoxanthine-guanine phosphoribosyltransferase (HPRT) given by the formula 2^-ΔCt^.

**Immunoblotting**

Following RNA preparation (see above), total protein was precipitated from the flow through of the RNA spin column by adding an equal volume of 100 mM ZnCl_2_, washed with cold acetone, and dissolved in 8 M urea containing 50 mM dithiothreitol. Protein concentration was determined by using the Bio-Rad Bradford protein assay kit (Bio-Rad, Hercules, CA, USA). Proteins were separated by SDS-PAGE and transferred to nitrocellulose membrane. The membranes were blocked in 5% skim milk powder (Fluka, Buchs, Switzerland), dissolved in TBST (50 mM TRIZMA base, 150 mM NaCl, pH 7.5 adjusted with HCl, 0.1% Tween-20), for one hour at room temperature. The membranes were then incubated over night at 4°C with the following primary antibodies: anti-cleaved Caspase-3 (#9664, Cell Signaling Technology, Boston, USA), anti-Lcn2 (a kind gift of M. Nilsen-Hamilton, Iowa State University, Ames, USA).([8](#_ENREF_8)) Following incubation of the membranes for one hour in HRP-conjugated secondary antibodies (diluted in 5% skim milk), immunocomplexes were visualized by ECL Western blotting detection reagents (Amersham, Buckinghamshire, UK). Relative intensities of the signals were quantified by densitometric scanning using a standard Image J program (NIH, Bethesda, MD) and normalized against loading controls.

**Assessment of renal function**

Mouse renal allograft function was assessed by serum creatinine, urea and Lcn2 measurements. Blood samples (0.4 - 0.6 ml) were taken at the time of sacrificing the animals from the inferior vena cava and centrifuged (10800 x g for 4 min) to isolate serum. Serum creatinine and urea were routinely measured using CREP2-Creatinine plus ver. 2 and UREAL, respectively on Roche/Hitachi Cobas c 701/702 systems (Roche Diagnostics, Mannheim, Germany) at the Central Institute for Medical and Chemical Laboratory Diagnostics (ZIMCL) of the Innsbruck Medical University (MUI). Serum levels of mouse Lipocalin-2/NGAL were determined by using Quantikine ELISA kit (R&D Systems, Minneapolis, MN).

**Histopathology, immunohistochemistry and TUNEL staining**

All kidney samples were fixed in 4.5% neutrally buffered formaldehyde for 18-24 hours prior to further processing. Dehydration, paraffin embedding and preparation of slides were done following standard histological procedures. Tissue slides were stained with Hematoxylin-Eosin (HE) and Periodic Acid Schiff’s stain (PAS). For histology tissue sections (4 µm) were stained with Hematoxylin-Eosin (HE) or Periodic Acid Schiff’s (PAS) stain and lesions were scored according to the definitions of Banff classification ([4](#_ENREF_4)). In addition to the defined lesions we also semiqunatitatively graded peri-arterial lymphoctic aggregates (PALA) (0: absent, grade 1/mild: less prominent PALA visible at intermediate to high magnification, grade 2/moderate: intermediate to grade 1 and grade 3, grade 3/severe: numerous prominent PALA around arteries visible at scanning view) and acute tubular injury (0: absent, grade 1/mild: tubular ectasia and focal cell detachment, grade 2/moderate: intermediate to grade 1 and grade 3, grade 3/severe: overt tubular epithelial cell necrosis involving entire tubular cross sections).

For immunohistochemical staining, 5 µm sections were mounted on Superfrost Plus slides (Menzel, Braunschweig, Germany), dewaxed 4 x 3 min in xylol, 1 x 3 min each in 100% ethanol, 96% ethanol, and 80% ethanol, rinsed for 5 min with water, and autoclaved for 10 min at 121°C in 10 mM sodium citrate pH 6.0 for antigen retrieval. Endogenous peroxidase activity was blocked by incubation in 1% H_2_O_2_ for 15 min, endogenous biotin was blocked employing the Biotin Blocking System (Dako, Glostrup, Denmark), and non-specific protein binding sites were blocked by incubation in TNB (TBS containing 0.5% Blocking Reagent, PerkinElmer, Rodgau, Germany) for 30 min. Slides were incubated for 16 h at 4°C with the following primary antibodies diluted in TNB: anti-Lipocalin 2 (provided by M. Nilsen-Hamilton, Iowa State University, Ames, USA; used at 1:2,000), anti-Cleaved Caspase-3 (#9664, Cell Signaling Technology, Boston, USA; used at 1:400), anti-CD3 (A-0452, Dako, Glostrup, Denmark; used at 1:400), anti-CD4 (SAB4503583, Sigma-Aldrich, St. Louis, USA; used at 1:300), anti-CD8 (bs-0648R, Bioss, Woburn, USA; used at 1:100), anti-mouse Gr-1 (MAB1037, R&D Systems, Minneapolis, USA; used at 1:600), followed by incubation for 2 h at 25°C with suitable horseradish peroxidase-conjugated immunoglobulins (Sigma-Aldrich, St. Louis, USA). The Tyramide Signal Amplification System (PerkinElmer, Rodgau, Germany) was used according to manufacturer's instructions for signal amplification. For staining of immunocomplexes, slides were incubated for 5 min with DAB substrate (0.05% 3,3'-diaminobenzidine, 0.01% H_2_O_2_, 50 mM Tris.HCl, pH 7.6) and counterstained with Mayer's hemalum (Merck, Darmstadt, Germany). Slides were dehydrated by incubation for 3 min each in 70% ethanol, 80% ethanol, 96% ethanol, 2 x 100% ethanol, and 2 x xylol and coverslips were mounted with Entellan (Merck, Darmstadt, Germany). For each slide, the number of immuno-stained cells was determined in three non-overlapping high power fields (400x magnification) and the mean value was calculated for comparison. TUNEL (terminal transferase-mediated dUTP nick end labeling) was performed using the In Situ Cell Death Detection Kit POD (Roche, Vienna, Austria) according to manufacturer's instructions. Briefly, slides were dewaxed as described above and tissues were pretreated for 15 min with 10 µg/ml Proteinase K and for 2 min in 0.1% Triton X-100. After blocking endogenous peroxidase for 15 min with 1% H_2_O_2_, sections were incubated for 60 min at 37°C with TUNEL-Mix followed by incubation for 30 min at 37°C with Converter-POD. Slides were developed for 5 min with DAB substrate, counterstained with Mayer's hemalum, dehydrated, and coverslipped. The mean value of the number of TUNEL positive nuclei counted in three non-overlapping high power fields (400x) was calculated for each slide.

**Statistical Analysis**

Statistical analyses were performed using the IBM SPSS Statistics 21 software package (IBM, Armonk, NY, USA). Group differences for scale data such as cell counts or expression levels were analyzed by the Kruskal-Wallis test and differences between individual groups of animals were determined using the Mann-Whitney test. Group differences for ordinal data such as pathological Banff scores were analyzed with the Chi-Square test. A p-value lower than 0.05 was considered statistically significant. Scale data for different parameters are shown as boxplots with medians, interquartiles, and ranges as provided by the software.

**Supplemental Figures**

**Figure S1: Histology of the renal grafts.** Kidneys were transplanted syngenically from C57Bl/6 (Bl/6) to Bl/6 mice and allogenically from Bl/6 to Balb/c and Balb/c to Bl/6 mice. The grafts were harvested at post tx day 7, stained with Hematoxylin-Eosin (HE) or Periodic Acid Schiff’s (PAS) and analysed for acute tubular injury (ATI) (A) and cast formation (B). The representative boxplots are shown.

References:

1. Rong S, Lewis AG, Kunter U, Haller H, Gueler F. A knotless technique for kidney transplantation in the mouse. Journal of transplantation 2012;2012:127215.

2. Hraba-Renevey S, Turler H, Kress M, Salomon C, Weil R. SV40-induced expression of mouse gene 24p3 involves a post-transcriptional mechanism. Oncogene 1989;4(5):601-608.

3. Ashraf MI, Ebner M, Wallner C, Haller M, Khalid S, Schwelberger H et al. A p38MAPK/MK2 signaling pathway leading to redox stress, cell death and ischemia/reperfusion injury. Cell communication and signaling : CCS 2014;12:6.

4. Haas M, Sis B, Racusen LC, Solez K, Glotz D, Colvin RB et al. Banff 2013 meeting report: inclusion of c4d-negative antibody-mediated rejection and antibody-associated arterial lesions. American journal of transplantation : official journal of the American Society of Transplantation and the American Society of Transplant Surgeons 2014;14(2):272-283.

5. Aigner F, Maier HT, Schwelberger HG, Wallnofer EA, Amberger A, Obrist P et al. Lipocalin-2 regulates the inflammatory response during ischemia and reperfusion of the transplanted heart. American journal of transplantation : official journal of the American Society of Transplantation and the American Society of Transplant Surgeons 2007;7(4):779-788.

6. Sickinger S, Maier H, Konig S, Vallant N, Kofler M, Schumpp P et al. Lipocalin-2 as mediator of chemokine expression and granulocyte infiltration during ischemia and reperfusion. Transplant international : official journal of the European Society for Organ Transplantation 2013;26(7):761-769.

7. Berger T, Togawa A, Duncan GS, Elia AJ, You-Ten A, Wakeham A et al. Lipocalin 2-deficient mice exhibit increased sensitivity to Escherichia coli infection but not to ischemia-reperfusion injury. Proceedings of the National Academy of Sciences of the United States of America 2006;103(6):1834-1839.

8. Liu Q, Ryon J, Nilsen-Hamilton M. Uterocalin: a mouse acute phase protein expressed in the uterus around birth. Molecular reproduction and development 1997;46(4):507-514.
